# The association between serum complement C3a and severity in patients with community-acquired pneumonia

**DOI:** 10.3389/fimmu.2023.1034233

**Published:** 2023-01-27

**Authors:** Zheng Xu, Xue-Feng Hou, Chun-Mei Feng, Ling Zheng, De-Xiang Xu, Hui Zhao, Lin Fu

**Affiliations:** ^1^ Department of Respiratory and Critical Care Medicine, Second Affiliated Hospital of Anhui Medical University, Hefei, Anhui, China; ^2^ Institute of Respiratory Diseases, Second Affiliated Hospital of Anhui Medical University, Hefei, Anhui, China; ^3^ Department of Respiratory and Critical Care Medicine, The Sixth People’s Hospital of Fuyang, Anhui, China; ^4^ School of Pharmacy, Drug Research and Development Center, Wannan Medical College, Wuhu, Anhui, China; ^5^ Department of Toxicology, Anhui Medical University, Hefei, Anhui, China

**Keywords:** community-acquired pneumonia, complement C3a, pro-inflammatory cytokines, CAP severity score, biomarker

## Abstract

**Background:**

A few studies found that the complement system may be involved in the onset and progression of community-acquired pneumonia (CAP). However, the role of the complement system in CAP was obscure. The goal of this study was to analyze the association of serum complement C3a with CAP severity scores based on a cross-sectional study.

**Methods:**

All 190 CAP patients and 95 control subjects were enrolled. Demographic information and clinical data were extracted. Peripheral blood samples were collected on admission.

**Results:**

Serum complement C3a on admission was elevated in CAP patients compared with healthy subjects. The level of complement C3a was gradually elevated in parallel with CAP severity scores (CURB-65, CRB-65, PSI, SMART-COP, and CURXO). Complement C3a was positively correlated with blood routine parameters, renal function markers, and inflammatory cytokines in CAP patients. Furthermore, multivariate linear and logistic regression models found that serum complement C3a on admission was positively associated with CAP severity scores. Mechanistic research suggested that complement system inhibition alleviated *Streptococcus pneumoniae-*induced upregulation of IL-1β, TNF-α, IL-6, and CRP in MLE-12 cells.

**Conclusions:**

Serum complement C3a on admission is positively associated with the severity of CAP patients. Inhibiting complement system attenuates *S. pneumoniae-*elevated secretion of inflammatory cytokines in pulmonary epithelial cells, indicating that complement C3a is involved in the pathophysiology of CAP. Serum complement C3a may serve as an earlier diagnostic biomarker for CAP.

## Background

Community-acquired pneumonia (CAP) is a syndrome in which an acute infection of the lungs develops in patients who have not been hospitalized recently and have no regular access to the healthcare system (1). The Gram-positive bacterium *Streptococcus pneumoniae* and viruses are the most common cause of pneumonia in adults (2, 3). More and more studies have revealed that CAP is consistently the principal reason for morbidity and mortality all over the world (4). The overall incidence of CAP in adults is approximately 5.16 to 6.11 cases per 1,000 persons per year (5). CAP is the fourth leading cause of death in Taiwan in 2014. Additionally, CAP is responsible for an average of 4.5 million annual visits to hospitals, emergency departments, and outpatient clinics (6). Therefore, immediate and optimized treatment of CAP is helpful for reducing morbidity and mortality. Earlier diagnosis and recognition of disease severity are imperative for improving the prognosis of CAP patients.

The complement system is an integral component of the innate immune response to pathogens and an instigator of proinflammatory responses, which can activate danger signals and disturb tissue homeostasis (7, 8). The complement system contains approximately 50 proteins, with additional regulators and functional receptors for activation products being discovered as new molecules and biomarkers. Complement activation leads to the cleavage of several cascading proteins and contributes to the formation of complement fragments, such as C3a, C4b, C5a, and C5b (9, 10). Mounting lines of evidence have revealed that the complement system exerts a central role in acute or chronic inflammatory diseases. After complement system activation, complement fragments are released to diffuse and further induce inflammatory reactions (11, 12). More and more studies have demonstrated that the involvement of complement-driven networks in inflammatory conditions ranges from cutaneous, renal, and ocular to neuroinflammatory and neurodegenerative disorders (13–16).

Recent studies have found that the complement system is activated and exacerbated in acute lung injury through autophagy-mediated alveolar macrophage apoptosis (17). *In vitro* and *in vivo* experiments found that complement C5a is elevated in lung tissues under inflammatory reactions (18). Recent research found that complement fragments (C4, C5) are increased in patients with coronavirus disease 2019 (COVID-19) (19). Moreover, several studies revealed that partial complement fragments are elevated in CAP patients (20, 21). These results indicated that the complement system may be involved in the progression of CAP. However, the association between the complement system and severity was unclear. Moreover, the exact role of the complement system in CAP was obscure. Therefore, the main purpose of this study was to analyze the association between serum complement level and the severity of CAP through a cross-sectional study. Furthermore, the influence of complement inhibition on the inflammatory reaction was explored *in vitro* experiments.

## Methods

### Subjects

From May 2018 to June 2020, 190 CAP patients and 95 age- and sex-matched healthy controls were enrolled in the study from the Department of Respiratory and Critical Care Medicine in the Second Affiliated Hospital of Anhui Medical University, Hefei City, Anhui Province. Healthy volunteers were from the physical examination center in the Second Affiliated Hospital of Anhui Medical University. Oral agreement or a consent form was gained from the patients or the patients’ next of kin. All CAP patients must meet the following criteria: more than 18 years old, confirmed with CAP, and subsequently hospitalized for CAP (22–24). Additionally, nucleic acid testing and chest x-rays were performed before hospitalization, and patients with COVID-19 were excluded. The exclusion criteria included the following: immunodeficiency diseases, suffered from infectious diseases in the past 3 months, rheumatic disease, patients with malignancies, pregnant women, and immunosuppressive or cytokine antagonists in the past 6 months. The clinical severity was evaluated by CAP severity scores. The current CAP severity scores were commonly used to assess the severity of CAP including CURB-65, CRB-65, PSI, SMART-COP, and CURXO scores. These CAP severity scores have their own characteristics and are widely used to evaluate the severity of CAP in clinical work. Assessing the severity of CAP is a key factor in developing a treatment plan. The clinical characteristic, demographic information, laboratory results, and comorbidities of CAP patients were collected from the medical record on admission. Demographic information of matched healthy cases was also collected from the physical examination center. The Institutional Ethics Committee of the Second Affiliated Hospital of Anhui Medical University approved this study (YX2021-147), which met the principles of the Declaration of Helsinki.

### Enzyme-linked immunosorbent assay

Serum samples were collected from participants in the morning and centrifugated (25). Inflammatory cytokine ELISA kits (TNF-α and IL-1β) from Wuhan Colorful Gene Biological Technology Co., Ltd. (http://www.jymbio.com/) were prepared. C3a, C4b, and C5a kits were purchased from Wuhan Cusabio Biotech Co., Ltd. (http://www.cusabio.cn/). All detection processes were conducted based on the manufacturer’s instructions (26, 27).

### Cell culture

MLE-12 (mouse origin) is an immortalized cell line of pulmonary epithelial cells. The MLE-12 cell line was obtained from the American Type Culture Collection (ATCC; Manassas, VA, USA). MLE-12 cells were grown in DMEM with 10% fetal bovine serum in a humidified chamber at 37˚C with 5% CO_2_ (28, 29). *Streptococcus pneumoniae* (*SP*) strain NCTC 7466 (D39, serotype 2) was bought from the National Collection of Type Cultures (NCTC; London, United Kingdom). In order to explore the possible mechanism, the complement C3 inhibitor compstatin Cp40 was used in this experiment. Cp40 is a very common complement C3a inhibitor. Cp40 is a common protein–protein interaction inhibitor; it inhibits complement activation by binding native C3 and its bioactive fragments C3b and C3c. In addition, Cp40 interferes with convertase formation and inhibits the binding and cleavage of native C3 by C3 convertases (30, 31). Cp40 (CAS: 206645-99-0) was from Beijing Solarbio Science and Technology Co., Ltd. All cells were divided into four groups: Control (CTRL) group, Cp40 group, SP group, and Cp40+SP group. In the Cp40 and Cp40+SP groups, MLE-12 cells were pre-incubated with Cp40 (200 µM final concentration) at 48 h and 24 h before *SP* exposure. Before performing the succeeding experiments, serum-free media were used to culture MLE-12 cells for 24 h. Then, MLE-12 cells were co-cultured with *SP* (10^7^ CFU/ml) at different times in the SP and Cp40+SP groups. The doses of *SP* and Cp40 used were chosen to be similar to the previous studies (32, 33). In the control group, MLE-12 cells were co-cultured with DMSO. At 12 h and 24 h after *SP* exposure, the supernatant and cells were collected. The expression of C3a and inflammatory cytokines were measured in MLE-12 cells.

### Isolation of total RNA and reverse transcription-polymerase chain reaction

Total RNA from MLE-12 cells was extracted using TRI reagent as described previously (34). Quantitative RNA was used to synthesize cDNA with avian myeloid leukemia virus reverse transcriptase (Promega). The mRNA levels were determined followed by real-time RT-PCR with a Light Cycler 480 SYBR Green I kit (Roche Diagnostics). Every gene was detected in three replicates and three times. The primer sequences are shown in **Table 1**. *18S* was used as a control and the relative expression of target genes was calculated using the 2^−ΔΔCT^ method.

**Table 1:** Oligonucleotide sequences and size of primers.

Genes	Sequences	Sizes (bp)	Species
*18S*	Forward: 5’-GTAACCCGTTGAACCCCATT-3’	109	Mouse
	Reverse: 5’-CCATCCAATCGGTAGTAGCG-3’		
*C3a*	Forward: 5’-TCCTTCACTATGGGACCAGC-3’	180	Mouse
	Reverse: 5’-GACTGGGATGTCACCCTGAG-3’		
*Crp*	Forward: 5’-TGGTGGGAGACATCGGAGAT-3’	105	Mouse
	Reverse: 5’-GCCCGCCAGTTCAAAACATT-3’		
*Tnf-α*	Forward: 5’-GATCGGTCCCCAAAGGGATG-3’	92	Mouse
	Reverse: 5’-CCACTTGGTGGTTTGTGAGTG-3’		
*Il-6*	Forward: 5’-TTCCTCTGGTCTTCTGGAGT-3’	144	Mouse
	Reverse: 5’-TGACTCCAGCTTATCTCTTGGTT-3’		
*Il-1β*	Forward: 5’-TGCCACCTTTTGACAGTGATG-3’	138	Mouse
	Reverse: 5’-TGATGTGCTGCTGCGAGATT-3’		

### Statistical analysis

All statistical analyses were performed using SPSS 19.0. All continuous variables were tested by the homogeneity of variance test and normality test. The continuous variables were shown with the mean or median. Classification variables were shown with frequency (percent). Student’s *t*-tests and Mann–Whitney *U* test were used to compare the demographic characteristics. The categorical variables were compared using Fisher exact test or *χ* ^2^ test. The correlations between serum complement C3a and clinical parameters were analyzed through the Spearman correlation coefficient or Pearson rank correlation in accordance with the normality test. Moreover, the associations between serum complement C3a and CAP severity scores were analyzed by multivariate linear and logistic regression models. In order to control confounding factors, age, sex, BMI, and comorbidities were adjusted. In the in vitro experiment, the expressions of inflammatory cytokines and C3a were compared using ANOVA or Student’s *t*-tests. Statistical significance was determined at *p* ≤ 0.05.

## Results

### Demographic data and clinical information

A total of 95 control subjects and 190 CAP patients were recruited. Pathogen detection was explored. As shown in **Table 2**, 67 (35.3%) patients were infected with *Streptococcus pneumoniae*, 15 (7.7%) with *Legionella pneumophila*, 36 (18.9%) with other atypical pathogens, 4 (2.1%) with a respiratory virus, 7 (3.7%) with *Pseudomonas aeruginosa*, and 61 (32.1%) with other pathogens. In addition, there was no difference in age, sex, body mass index (BMI), systolic pressure, diastolic pressure, and glucose in the two groups. Moreover, we found that alanine aminotransferase (ALT) and aspartate aminotransferase (AST) were increased in CAP patients. Direct bilirubin (DBIL) was higher and indirect bilirubin (IBIL) was lower in CAP patients. In addition, urea nitrogen and uric acid were higher in CAP patients. Furthermore, a blood routine biochemical examination was conducted. As shown in **Table 2**, the counts of white blood cells (WBCs) and neutrophils were elevated in CAP patients. Platelet–lymphocyte ratio (PLR), neutrophil–lymphocyte ratio (NLR), and monocyte–lymphocyte ratio (MON) were higher in CAP patients compared with healthy volunteers. Furthermore, inflammatory cytokines, mainly consisting of procalcitonin (PCT), tumor necrosis factor α (TNF-α), interleukin-1β (IL-1β), C-reactive protein (CRP), and interleukin-6 (IL-6), were elevated in CAP patients. Simultaneously, pneumonia severity was evaluated through CAP severity scores. There were 88 (46.3%) severe subjects in CAP patients (CURXO score). The average CURB-65, CRB-65, pneumonia severity index (PSI), and SMART-COP were 1.8, 1.5, 99.70, and 2.9, respectively (**Table 2**).

**Table 2:** Demographic and clinical characteristics between CAP patients and control subjects.

Variables	CAP (*n* = 190)	Control (*n* = 95)	p
*Streptococcus pneumoniae*, n (%)	67 (35.3)	N.A.	N.A.
*Legionella pneumophila*, n (%)	15 (7.7)	N.A.	N.A.
Other atypical pathogens, n (%)	36 (18.9)	N.A.	N.A.
Respiratory virus, n (%)	4 (2.1)	N.A.	N.A.
*Pseudomonas aeruginosa*, n (%)	7 (3.7)	N.A.	N.A.
Others, n (%)	61 (32.1)	N.A.	N.A.
Age (years)	66.8 ± 1.88	60.8 ± 2.35	0.452
Male, n (%)	108 (56.8)	50 (52.6)	0.321
BMI	21.6 (19.0, 23.8)	21.3 (19.2, 24.3)	0.221
Systolic pressure (mmHg)	122.7 (99.8, 140.6)	120.1 (103.5, 139.5)	0.564
Diastolic pressure (mmHg)	72.6 (55.6, 82.6)	78.0 (60.5, 84.6)	0.521
Glucose (mmol/L)	5.45 ± 0.13	5.05 ± 0.16	0.324
ALT (U/L)	28.2 ± 2.70	18.7 ± 3.03	<0.05
AST (U/L)	31.9 ± 2.22	20.1 ± 1.62	<0.01
TBIL (μmol/L)	13.3 ± 0.98	16.1 ± 1.58	0.098
DBIL (μmol/L)	4.01 ± 0.64	2.75 ± 0.25	<0.01
IBIL (μmol/L)	9.24 ± 0.50	13.35 ± 1.34	<0.05
Urea nitrogen (mmol/L)	6.04 ± 0.35	4.36 ± 0.32	<0.05
Creatinine (μmol/L)	68.2 ± 4.78	61.2 ± 4.66	0.105
Uric acid (μmol/L)	279.2 ± 11.59	374.2 ± 35.82	<0.01
WBC (10^9^/L)	8.3 ± 0.55	5.7 ± 0.32	<0.01
Neutrophil (10^9^/L)	6.95 ± 0.84	3.1 ± 0.27	<0.01
Lymphocyte (10^9^/L)	2.44 ± 0.92	2.05 ± 0.14	0.458
NLR	6.6 ± 0.73	1.6 ± 0.18	<0.01
MON	0.4 ± 0.04	0.2 ± 0.06	<0.01
PLR	229.2 ± 15.32	123.7 ± 18.86	<0.01
PCT (ng/L)	0.1 ± 0.03	1.8 ± 0.21	<0.01
TNF-α (pg/ml)	738.5 ± 52.89	88.4 ± 7.85	<0.01
IL-1β (pg/ml)	475.3 ± 43.8	44.9 ± 3.85	<0.01
IL-6 (pg/ml)	206.23 ± 94.0	35.4 ± 12.31	<0.01
CRP (mg/L)	57.9 ± 7.34	12.0 ± 1.5	<0.01
CURB-65	1.8 ± 0.16	N.A.	N.A.
CRB-65	1.5 ± 0.13	N.A.	N.A.
PSI	99.7 ± 5.15	N.A.	N.A.
CURXO [severe, n (%)]	88 (46.3)	N.A.	N.A.
SMART-COP	2.9 ± 0.28	N.A.	N.A.

Not available, N.A.

### The level of serum complement C3a in control and CAP subjects

**Figure 1:**
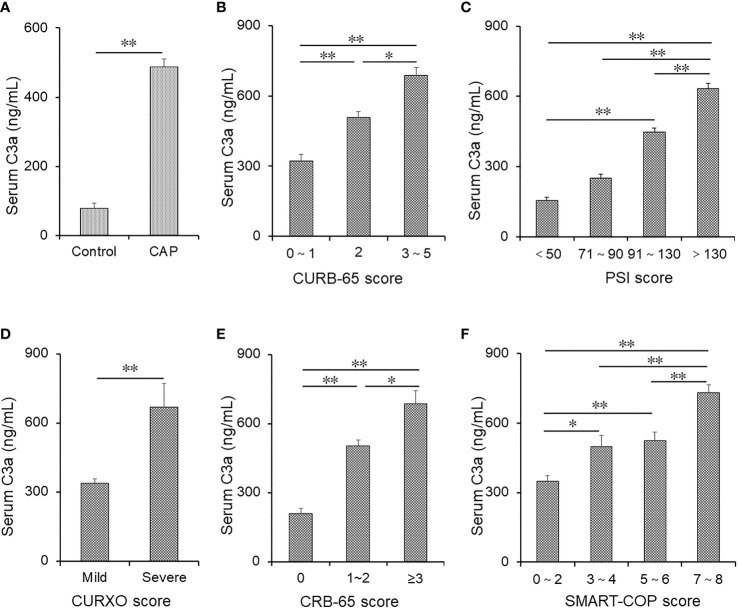
The levels of serum C3a in patients with CAP and control subjects. **(A–F)** Serum C3a was detected using ELISA in CAP patients and control subjects. **(A)** The levels of serum C3a were compared between control subjects (*n* = 95) and CAP patients (*n* = 190). **(B)** The levels of serum C3a were measured in patients with different CURB scores (score of 0–1: *n* = 95; score of 2: *n* = 56; score of 3–5: *n* = 48). **(C)** The levels of serum C3a were measured in patients with different PSI scores (score of <50: *n* = 34; score of 71–90: *n* = 22; score of 91–130: *n* = 32; score of >130: *n* = 102). **(D)** The levels of serum C3a were compared in patients with different CURXO scores (mild: *n* = 112; severe: *n* = 78). **(E)** The levels of serum C3a were compared in patients with different CRB-65 scores (score of 0: *n* = 44; score of 1–2: *n* = 108; score of ≥3: *n* = 38). **(F)** The levels of serum C3a were compared in patients with different SMART-COP scores (score of 0–2: *n* = 81; score of 3–4: *n* = 51; score of 5–6: *n* = 24; score of 7–8: *n* = 34). Data were expressed as the mean ± SEM. The statistical difference was analyzed by ANOVA or Student’s *t*-tests. *p < 0.05, **p < 0.01.

The level of serum complement C3a was determined between the two groups. As shown in **Figure 1A**, complement C3a was higher in CAP patients compared with healthy volunteers. Moreover, we found that serum C3a was lower in those with a score of 0–1 than others based on the CURB-65 score. The level of serum C3a was lower in those with a score of 2 than in those with a score of 3–5 (**Figure 1B**). In addition, serum C3a was lowest in those with a score of <50 and highest in those with a score of >130 in accordance with the PSI score (**Figure 1C**). According to the CURXO score, serum C3a was elevated in severe patients with CAP (**Figure 1D**). Moreover, serum C3a was gradually increased on the basis of the CRB-65 score (**Figure 1E**). Finally, the level of serum C3a was compared among CAP patients with different SMART-COP scores. As shown in **Figure 1F**, serum C3a was lower in those with a score of 0–2 than in those with a score of 3–4 and 5–6. The level of complement C3a was highest in those with a score of 7–8. Meanwhile, the level of serum C3a was compared in CAP patients with different pathogens. However, no significant difference in complement fragments was observed in CAP patients with different pathogens (data not shown). Moreover, the levels of C4b and C5a were detected. As shown in **Supplementary Table 1**, we found that the levels of C4b and C5a were all gradually elevated in parallel with the severity scores in CAP patients.

### Correlations of serum complement C3a with clinical parameters

The correlations between routine blood parameters and complement C3a were determined in CAP patients. As shown in **Table 3**, complement C3a was positively associated with WBC (*r* = 0.221; *p* = 0.029) in CAP patients. In addition, there were significantly positive correlations between complement C3a with NLR (*r* = 0.323; *p* = 0.001), MON (*r* = 0.279; *p* = 0.005), and PLR (*r* = 0.293; *p* = 0.003) among CAP patients. No statistical correlation between complement C3a and liver functional parameters was observed in CAP patients (**Table 3**). Moreover, there was a positive correlation between complement C3a and urea nitrogen (*r* = 0.394; *p* < 0.001) (**Table 3**). Furthermore, correlative analyses found that complement C3a was positively related to inflammatory cytokines, such as TNF-α (*r* = 0.629; *p* < 0.001), IL-1β (*r* = 0.549; *p* < 0.001), PCT (*r* = 0.345; *p* = 0.011), and CRP (*r* = 0.319; *p* = 0.005) (**Table 3**).

**Table 3:** Correlations between serum C3a and clinical parameters.

Variables	WBC	Neutrophil	NLR	MON	PLR
*R*	0.221	0.077	0.323	0.279	0.293
*P*	0.029	0.450	0.001	0.005	0.003
Variables	ALT	AST	Creatinine	Uric acid	Urea nitrogen
*R*	−0.173	−0.011	0.197	−0.148	0.394
*P*	0.088	0.913	0.052	0.146	<0.001
Variables	PCT	TNF-α	IL-1β	IL-6	CRP
*R*	0.345	0.629	0.549	0.238	0.319
*P*	0.011	<0.001	<0.001	0.090	0.005

### Associations of serum complement C3a with CAP severity scores

The associations between complement C3a and CAP severity scores were analyzed by multivariate linear and logistic regression models. As shown in **Table 4**, age, sex, BMI, and comorbidities were adjusted. Multivariate linear regression model indicated that there were positive associations between complement C3a with CURB-65 [odds ratio (OR) = 1.007; 95% CI: 1.001, 1.012], CRB-65 (OR = 1.113; 95% CI: 1.003, 1.512), PSI (OR = 1.121; 95% CI: 1.004, 1.415), and SMART-COP (OR = 1.006; 95% CI: 1.003, 1.210) scores among CAP patients. In addition, CAP patients were divided into three grades based on the levels of serum complement C3a by tertiles. Tertile 1 group, <337.6 ng/ml; Tertile 2 group, 337.6–602.1 ng/ml; Tertile 3 group, >602.1 ng/ml. The multivariate logistic regression model found that compared with Tertile 1, the ORs of severity scores in Tertile 3 were all elevated. Not only that, serum complement C3a on admission was positively associated with CAP severity scores (all *p* trend < 0.05).

**Table 4:** Associations between serum C3a and CAP severity scores.

Variables	Estimated changes by continuous serum C3a	Estimated changes (95% CI) by tertiles of serum C3a			*p* trend
		Tertile 1 (<337.6 ng/ml)	Tertile 2 (337.6–602.1 ng/ml)	Tertile 3 (>602.1 ng/ml)	
N	190	63	64	63	
CURB-65	1.007 (1.001, 1.012)	0 (Ref)	1.782 (0.390, 8.132)	**16.361 (4.083, 65.560)**	<0.001
CRB-65	1.113 (1.003, 1.512)	0 (Ref)	1.000 (0.187, 5.344)	**8.510 (2.141, 33.830)**	<0.001
PSI	1.121 (1.004, 1.415)	0 (Ref)	3.179 (0.764, 13.228)	**8.510 (2.141, 33.830)**	<0.001
SMART-COP	1.006 (1.003, 1.210)	0 (Ref)	3.125 (0.871, 11.214)	**10.385 (2.934, 37.751)**	0.003
CURXO	1.011 (0.998, 1.016)	0 (Ref)	2.774 (0.844, 9.122)	**20.714 (5.840, 73.479)**	0.041

Dependent variable: C3a. Independent variables: CURB-65, CRB-65, PSI, SMART-COP, and CURXO.

*Adjusted for age, sex, BMI, and comorbidities. Data in bold denote statistically significant results.

### Predictive capacities for severity between complement C3a and CAP severity scores

**Figure 2:**
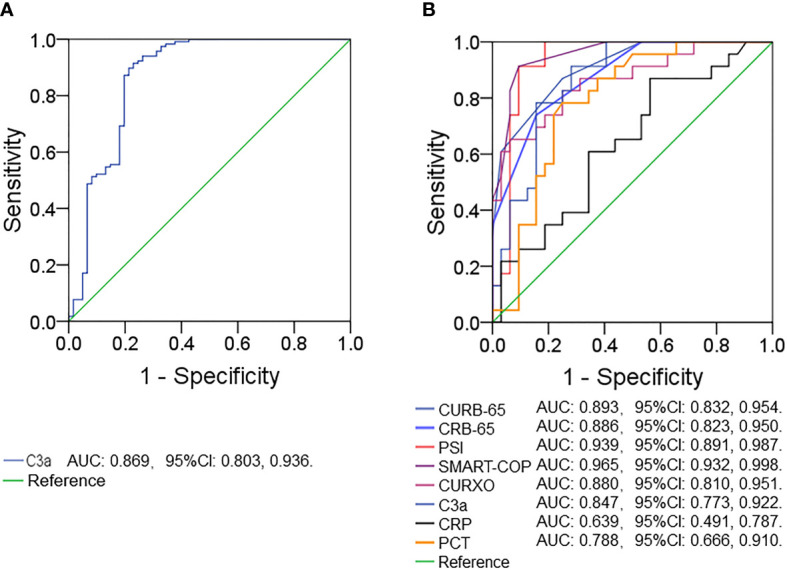
Receiver operating characteristic curves for different predictive indexes on admission. **(A)** ROC curve was used to evaluate the predictive value of serum complement C3a for CAP. **(B)** ROC curve was used to evaluate the predictive values of different indexes (C3a, CRB-65, CURB-65, CURXO, SMART-COP, PSI, CRP, and PCT) for the CAP severity. Receiver operating characteristic, ROC.

The predictive capacities for CAP were explored by receiver operating characteristic (ROC) area under the curve (AUC) analysis. As shown in **Figure 2A**, the AUC of serum complement C3a for CAP prediction was 0.869 (95% CI: 0.803, 0.936). Furthermore, the AUCs for severity prediction were analyzed among CAP patients. As shown in **Figure 2B**, the AUC of C3a for severity was 0.847 (95% CI: 0.773, 0.922). Meanwhile, the levels of AUCs between complement C3a and CAP severity scores with inflammatory cytokines were compared. The results suggested that AUCs of CURB-65, CRB-65, PSI, SMART-COP, CURXO, PCT, and CRP for severity were 0.893 (95% CI: 0.832, 0.954), 0.886 (95% CI: 0.823, 0.950), 0.939 (95% CI: 0.891, 0.987), 0.965 (95% CI: 0.932, 0.998), 0.880 (95% CI: 0.810, 0.951), 0.788 (95% CI: 0.810, 0.951), and 0.639 (95% CI: 0.491, 0.787), respectively (**Figure 2B**).

### Inhibiting complement system attenuated *SP*-evoked inflammatory cytokines in MLE-12 cells

**Figure 3:**
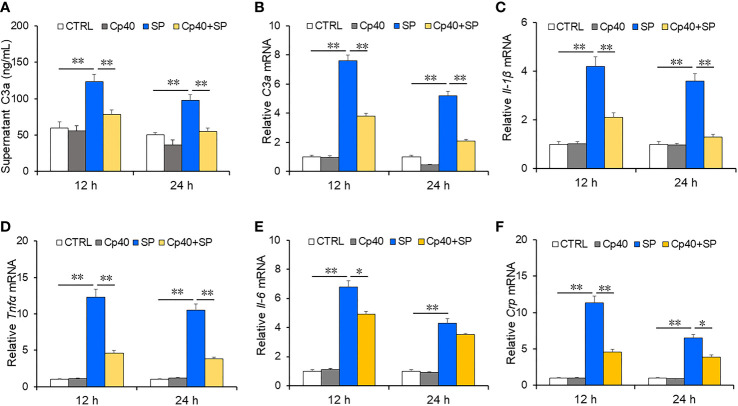
The levels of complement C3a and inflammatory cytokines in MLE-12 cells after *SP* infection. In the Cp40 and Cp40+SP groups, MLE-12 cells were pre-incubated with Cp40 (200 µM) at 24 h and 48 h before SP exposure. Then, MLE-12 cells were exposed with SP (10^7^ CFU/ml) in SP and Cp40+SP groups. In the control group, MLE-12 cells were co-cultured with DMSO. Lastly, supernatant and cells were collected at 12 h and 24 h after SP exposure. The expressions of C3a and inflammatory cytokines were measured in MLE-12 cells. **(A)** The level of supernatant complement C3a was measured at 12 and 24 h after SP injection in MLE-12 cells through ELISA. **(A)** C3a. **(B)** The level of complement C3a mRNA was measured through RT-PCR at 12 and 24 h after SP infection in MLE-12 cells. **(B)** C3a mRNA. **(C–F)** The levels of inflammatory cytokine mRNAs were measured through RT-PCR at 12 and 24 h after SP injection in MLE-12 cells. **(C)** IL-1β mRNA; **(D)** TNF-α mRNA; **(E)** IL-6 mRNA; **(F)** Crp mRNA. All data were expressed as mean ± SEM of six samples (*n* = 6). The statistical difference was analyzed by ANOVA method. *p < 0.05, **p < 0.01.

To further explore the role of complement C3a among CAP patients, MLE-12 cells were pre-treated with complement system inhibitor and then exposed with *SP.* The expressions of pro-inflammatory cytokines and complement C3a were measured using ELISA and RT-PCR. As expected, simple CP40 pre-treatment had no obvious effect on the level of C3a compared with the control group in MLE-12 cells (**Figures 3A, B**). Complement C3a was increased at 12 h and 24 h after *SP* exposure. Interestingly, pre-treatment with CP40 significantly inhibited *SP*-induced the upregulation of complement C3a in 12 h and 24 h after *SP* exposure (**Figures 3A, B**). Furthermore, pro-inflammatory cytokines were measured in MLE-12 cells. There was no significant difference in pro-inflammatory cytokines between the control group and the CP40 group. However, mRNAs of pro-inflammatory cytokines were dramatically elevated at 12 h and 24 h after *SP* exposure (**Figures 3C–F**). Although CP40 pre-treatment had no effect on *SP*-induced secretion of inflammatory cytokines (**Figure 3E**), it significantly attenuated *SP*-induced elevation of *Il-1β, Tnf-α, Il-6*, and *Crp* mRNAs in MLE-12 cells.

## Discussion

This was the first epidemiological and laboratory research to explore the association of the complement system with severity scores among CAP patients. The major findings of this research include the following: Firstly, serum complement C3a is elevated in CAP patients; secondly, serum complement C3a is positively associated with CAP severity scores; thirdly, there are similar predictive capacities for severity between serum complement C3a and CAP severity scores; and fourthly, inhibiting the complement system attenuates *SP*-evoked secretion of inflammatory cytokines in MLE-12 cells.

Mounting lines of evidence suggested that the complement system is involved in the occurrence and development of inflammatory diseases. Previous studies have found that different complement fragments are increased in patients with acute lung injury, lung inflammation, and COVID-19 (17–19). In addition, our previous studies have demonstrated that inflammatory reaction is elevated in CAP patients (35–38). Nevertheless, the associations of the complement system with CAP severity were unclear. In this research, serum complement C3a was increased in CAP patients compared with healthy subjects. Moreover, serum complement C3a was gradually elevated with increased CAP severity scores. Multivariate linear and logistic regression models revealed that serum complement C3a was positively associated with CAP severity scores. These results provide lines of evidence that the level of serum complement C3a is positively associated with the severity of CAP.

Several studies found that pneumonia-induced pulmonary hyperpermeability can be attributed to both direct effects of pathogenic factors and an uncontrolled host response. In host factors, neutrophil recruitment increases the release of proinflammatory cytokines, which may contribute to pathogenesis (39, 40). Pro-inflammatory cytokines are increased in CAP patients (41). The complement system is best known as a regulator of inflammation and immune clearance of pathogens (42). Consequently, the associations between pro-inflammatory cytokines and serum complement C3a were analyzed. We found that pro-inflammatory cytokines were elevated in CAP patients. Serum complement C3a was positively associated with pro-inflammatory cytokines. Additionally, an earlier study indicated that the change in routine blood indexes is associated with unfavorable clinical outcomes in CAP patients (43). The associations of blood routine indexes with serum complement C3a were investigated in CAP patients. The results indicated that complement C3a was weakly and positively correlated with blood routine indexes (WBC, NLR, MON, and PLR). Moreover, serum complement C3a was positively associated with renal dysfunction. These results indicate that the serum complement system may be involved in the pathophysiology of CAP.

A good association between the serum complement system and CAP severity scores suggested that it may be a predictive biomarker in CAP patients. The predictive power was analyzed using the ROC curve. The AUC value of complement C3a for CAP was 0.869. Furthermore, our results demonstrated that there were similar predictive powers in AUC values for CAP severity between serum complement C3a and CAP severity scores. In addition, our research revealed that the predictive powers of serum complement C3a for severity were elevated compared with CRP and PCT in CAP patients. Moreover, serum complement C3a was easily and inexpensively detected. Therefore, serum complement C3a may be regarded as a more convenient and dominant diagnostic biomarker for CAP.

At present, although the associations of complement fragments with inflammatory diseases have made some progress, the specific molecular mechanisms are still not well clarified. The complement system not only acts as a sensor of pathogens but also recognizes diseased and damaged host cells. It closely collaborates with other immune and defense systems to eliminate potential danger and mediate inflammatory responses (44). The hub-like organization of the complement and its cell surface-directed action are essential for regulating the complement response to different triggers (45). When suffering from foreign pathogens, host cells can induce distinct initial pathways, mainly including classical pathways, lectin pathways, and alternative pathways. After complement C3 converges, the aim of antibodies and phagocytes is to clear microbial intruders (*via* C3b opsonization) and promote inflammation (*via* anaphylatoxins C3a and C5a). Lysed susceptible pathogens (*via* the C5b-9 membrane attack complex) are generated. Moreover, after complement C4 is activated, complement C3 convertase complexes are assembled and then cleave the abundant complement C3 into an anaphylatoxin fragment (C3a) and the opsonin C3b. Complement fragments are generated after activating the complement system. In the pre-antibiotic era, *SP* is the most common cause of pneumonia in adults. A previous study found that *SP* infection induced the upregulation of complement factors C1q and C3a in an *in vivo* experiment (46). Our study found that complement C3a was increased in *SP-*exposed MLE-12 cells. These results indicated that *SP* infection improved the generation of complement fragments in CAP patients. These complement fragments that bind to the toll-like receptor or complement receptor promote inflammation. The cross-talk between TLR and complement fragments not only are important for antimicrobial defense but also contributes to inflammatory disorders. Previous studies found that the formation of complement fragments would facilitate the secretion of pro-inflammatory cytokines (47, 48). A dramatic increase in inflammatory cytokines is involved in the pathophysiology of pneumonia (49). Additionally, C1q- and C3-deficient mice infected with *SP* reduced inflammatory cytokine secretion (50). In the present study, we found that pro-inflammatory cytokines and complement C3a were increased in the pulmonary epithelial cells after *SP* exposure. Interestingly, inhibiting the complement system significantly alleviated *SP*-evoked inflammatory cytokines. These results suggest that *SP* injection induced pneumonia, at least partially, through a complement system-mediated inflammatory reaction.

This study further reviewed the role of the complement system in CAP. There were some merits in the current research. We found that the level of serum C3a level on admission was positively associated with the severity scores in CAP patients. Inhibiting the complement system alleviated *SP*-induced inflammatory cytokine secretion in pulmonary epithelial cells. However, we also acknowledged some shortcomings. Firstly, this study had a small sample size and was a single-center research study; thus, a study with a larger sample size that involves multicenter research needs to be carried out in the future. Only in this way can one guarantee the results’ reliability and representativeness. Secondly, complement-activating products have more than 50 kinds of proteins. Nevertheless, due to the limitation in the experiment’s progress and for practical purposes, only serum C3a was detected in the present research. The associations between more complement products and severity will be explored in succeeding work. Thirdly, because of the difference in manufacturers, brands, and cell lines, the applied concentration of CP40 (200 μM) is higher than that in other experiments. More *in vitro* experiments are required to confirm this.

## Conclusions

In brief, the present study mainly investigated the association between serum complement C3a and the severity of CAP patients based on a cross-sectional study. These results indicate that complement C3a is increased in CAP patients compared with healthy volunteers. The level of complement C3a is gradually increased in parallel with the severity of CAP. Serum complement C3a is positively associated with CAP severity scores. Complement system inhibition protects the *SP-*induced secretion of inflammatory cytokines in pulmonary epithelial cells. These results firstly provide lines of evidence that the complement system may exert an important role in the pathophysiology of CAP. Consequently, serum complement C3a may be a diagnostic biomarker for CAP patients in clinical management.

## Data availability statement

The original contributions presented in the study are included in the article/**Supplementary Material**. Further inquiries can be directed to the corresponding authors.

## Ethics statement

The studies involving human participants were reviewed and approved by the Ethics Committee in Second Affiliated Hospital of Anhui Medical University and met the declaration of Helsinki. The patients/participants or patients/patients’ next of kin provided their written informed consent to participate in this study.’

## Author contributions

LF and HZ conceived the study. LF, HZ, ZX, X-FH, C-MF, LZ, and D-XX participated in the design of this study. LF and HZ obtained research funding, provided reagents, and supervised the conduct of the study. ZX, X-FH, LZ, C-MF, and LF conducted the research. LF and ZX conducted statistical analyses of all data. ZX drafted the manuscript. All authors contributed to the article and approved the submitted version.
