# Supplementary material for: The association between serum complement C3a and severity in patients with community-acquired pneumonia
Source: Front Immunol. 2023 Jan 27;14:1034233. doi: 10.3389/fimmu.2023.1034233 (PMC9911530; doi:10.3389/fimmu.2023.1034233)
Supplement: Supplementary file 1 [file Table_1.docx]

Supplemental Table 1. The levels of complement system between CAP patients and Control subjects.

| Characteristic |  | N | C4b (ng/mL) | C5a (pg/mL) |
| --- | --- | --- | --- | --- |
| Control |  | 190 | 205.5±12.08 | 402.8±48.44 |
| CAP |  | 95 | 488.9±14.90^††^ | 1298.8±44.48^††^ |
| CURB-65 |  |  |  |  |
|  | 0-1 score | 90 | 425.6±20.70 | 1071.1±62.79 |
|  | 2 score | 48 | 547.9±22.40* | 1451.6±43.97** |
|  | 3-5 score | 52 | 585.9±29.53** | 1541.3±78.47** |
| CRB-65 |  |  |  |  |
|  | 0 score | 50 | 349.9±22.50 | 822.8±64.84 |
|  | 1-2 score | 102 | 539.9±14.38** | 1460.5±43.82** |
|  | ≥3 score | 38 | 631.5±29.55**^##^ | 1622.3±35.91**^#^ |
| PSI |  |  |  |  |
|  | ＜50 score | 54 | 288.9±15.78 | 621.2±39.96 |
|  | 71-90 | 23 | 418.3±15.90** | 1052.1±26.80** |
|  | 91-130 | 33 | 524.7±16.98**^#^ | 1419.3±34.49**^#^ |
|  | ＞130 | 80 | 599.7±15.74**^#^ | 1602.1±33.36**^†^ |
| CURXO |  |  |  |  |
|  | Mild | 112 | 436.1±16.79 | 1111.8±49.83 |
|  | Severe | 78 | 602.2±21.83** | 1603.1±55.39** |
| SMART-COP |  |  |  |  |
|  | 0-2 score | 118 | 438.9±16.41 | 1159.6±56.42 |
|  | 3-4 score | 12 | 562.1±39.72** | 1383.3±89.56* |
|  | 5-6 score | 25 | 600.8±25.60** | 1468.2±43.19** |
|  | 7-8 score | 35 | 652.6±31.59** | 1665.6±49.24**^#^ |

Compared with Control, ^††^*P*≤0.01.

CURB-65: Compared with 0 score grade, ***P*≤0.01; Compared with 1-2 score grade, ^##^*P*≤0.01.

CRB-65: Compared with 0 score grade, **P*≤0.05, ***P*≤0.01; Compared with 2 score grade, ^#^*P*≤0.05, ^##^*P*≤0.01.

PSI: Compared with ＜50 score grade, **P*≤0.05, ***P*≤0.01; Compared with 71-90 score grade, ^#^*P*≤0.05, ^##^*P*≤0.01; Compared with 91-130 score grade, ^†^*P*≤0.05, ^††^*P*≤0.01.

CURXO: Compared with mild patients, ^††^*P*≤0.01.

SMART-COP: Compared with 0-2 score grade, **P*≤0.05, ***P*≤0.01; Compared with 3-4 score grade, ^#^*P*≤0.05, ^##^*P*≤0.01;

Compared with 5-6 score grade, ^††^*P*≤0.01.
